# Discrimination and avoidance due to disability in Australia: evidence from a National Cross Sectional Survey

**DOI:** 10.1186/s12889-018-6234-7

**Published:** 2018-12-05

**Authors:** Jeromey B. Temple, Margaret Kelaher, Ruth Williams

**Affiliations:** 1 Demography and Ageing Unit, Centre for Health Policy, Melbourne School of Population and Global Health, University of Melbourne, 207 Bouverie St, Melbourne, VIC 3010 Australia; 2 Centre for Health Policy, Melbourne School of Population and Global Health, University of Melbourne, Melbourne, Australia

**Keywords:** Disabilities, Discrimination, Avoidance

## Abstract

**Background:**

Across most high-income countries, populations are ageing. With this demographic change is an increase in the number of people living with disabilities. In this context, we sought to examine the prevalence of disability discrimination and disability avoidance in Australia, the demographic and health correlates of exclusion and the contexts in which disability discrimination and avoidance are experienced. Methods: Utilising newly released measures from the 2015 ABS Survey of Disability, Ageing and Carers, we calculate the prevalence of people living with a disability who have experienced discrimination and engage in avoidance behaviours, and the contexts in which they occur. Logistic regression models were fitted to examine the correlates of discrimination and avoidance behaviours, once controls and complex survey design were accounted for.

**Results:**

Approximately 9% (95% CI = 8.1, 9.2) of people with a disability experienced disability discrimination in 2015 and 31% (95% CI = 30.9, 32.9) engaged in avoidance behaviours because of their disability. With controls included, the prevalence of avoidance and discrimination declined with age, was higher for divorced people (versus married), the unemployed (versus employed) and was lower for people with lower levels of education (versus a degree) and those born overseas. Having a psychosocial or physical disability significantly increased the odds of experiencing discrimination or avoidance, as did having an increasing number of long-term health conditions. We further find that disability discrimination and avoidance occurs in contexts critical to human capital, such as the workforce, education and healthcare.

**Conclusions:**

Despite protections in legislation and international accords, significant proportions of Australians with a disability experience discrimination or engage in avoidance behaviours in various settings with potentially important human capital implications. Recently, sectoral responses (eg., in education and the workplace) have been offered by Government reports, providing direction for future research and evaluation.

## Background

Across most high-income countries, the number of people living wth disability and comorbidities is expected to grow significantly [1, 2]. For example, in Australia it is estimated that the population of older Australians with a profound disability will increase by 70% from 2006 to 2031 [3]. In the UK, it is projected that an additional 40% of people of any age will be living with a disability over the 20 years to 2022 [4].

One policy challenge that this demographic change poses is how to provide for the healthcare needs of this group, particularly in the context of rising healthcare costs partially attributable to population ageing [5]. A further related challenge is to ensure the social and economic wellbeing of this growing population by ensuring social inclusion and freedom from discriminatory practices or exclusion from services due to the presence of disability [6]. In 2006, the UN established the Convention on the Rights of Persons with Disabilities (CRPD) which was the first legally binding international accord that obligates signatory states to provide equal access to healthcare and related services for people with disabilities. The foundations of the CRPD include respect for human dignity and non-discrimination, full participation, social inclusion, equality of opportunity, and accessibility [7]. Like many other countries, Australia ratified the CRPD in 2008, building upon protections previously enshrined in the Disability Discrimination Act 1992 which sought to ensure equal rights, opportunities and access for people with a disability.

Despite protections against disability discrimination afforded in legislation and international accords in Australia, there is a significant evidence base on the deleterious social and economic outcomes faced by those living with a disability. For example, those living with a disability in Australia fare poorly on a range of socio-economic indicators including education, employment, housing vulnerability and a range of financial wellbeing measures [8, 9]. Those with multiple and specific disabilities (physical or psychological disabilities) have been found to face barriers to accessing healthcare and importantly, experiencing a barrier to healthcare has been shown to be associated with low levels of trust in health professionals and perceptions of discrimination in healthcare settings [10]. In a further recent study, Temple and Kelaher (2018) show that experiencing discrimination or avoidance due to disability is associated with deleterious mental health through psychological distress [11]. Another Australian study of the working age population (aged 15–64 years) found disability discrimination increased the odds of psychological distress as well as poorer self-rated health [12]. Furthermore, reports of discrimination were found to be more common among those living in disadvantageous circumstances. The association between experiencing disability discrimination and poorer self-rated health has also previously been observed in Canada [13]. More generally, however, there is a lack of nationally representative empirical evidence on the prevalence and situations or contexts in which disability discrimination is experienced in Australia. To the authors’ knowledge, there are no multivariable studies of the likelihood of discrimination and avoidance specifically.

In this paper, we examine two complementary aspects of disability exclusion – discrimination and avoidance specifically due to a person’s disability. The Australian Bureau of Statistics (ABS) defines disability discrimination as occurring when people “felt they had been unfairly considered or treated due to their disability” [14]. Disability avoidance is defined as “not going or staying away from people or places because of one’s disability” [14]. These concepts of disability exclusion have been operationalised by the ABS in the 2015 Survey of Disability, Ageing and Carers (SDAC). In a brief infographic published by the ABS from these data, they show 1 in 12 people with disabilities experienced discrimination in the previous 12 months [15].

With the availability of new nationally representative data, we seek to answer three questions. Firstly, how does the prevalence of disability discrimination and disability related avoidance vary by demographic characteristics, once controls for health conditions are included? Secondly, how does the prevalence of disability discrimination and disability related avoidance vary by disability characteristics, once controls for demographic characteristics are included? Finally, in what contexts do people with a disability experience discrimination and engage in avoidance behaviours? We follow our analysis with a discussion of the relevance of our findings and point to areas of extension and future research.

## Methods

### Data

Data for this study are from the 2015 Survey of Disability, Ageing and Carers (SDAC) conducted between July and December 2015. Three populations were sampled using multi-stage sampling techniques. These consisted of people living in private dwellings, in self-care retirement villages and in care accommodation. Within these populations, the ABS sought to collect information from people with a disability, individuals aged 65 years and older, and carers of people with a disability or long-term health condition.

This study utilises information collected from the household component of the survey, which includes individuals living in private residences and self-care retirement villages. Of 31,957 private dwellings contacted, 25,555 fully responded, yielding a response rate of 80%. Of the 288 people in self-care retirement villages contacted, 251 responded yielding a response rate of 87.2%. The corresponding response rate for care-accommodation was 89.4%. The module on discrimination and avoidance was included in a personal interview for all people aged 15 years and over with a disability who live in households. This reduced the sample size for analysis to 9763.

### Measures

For the first time in 2015, the ABS included a module on disability discrimination and disability avoidance. Firstly, respondents were asked “In the last 12 months do you feel that you have experienced discrimination or have been treated unfairly by others because of your condition/s?” For those who responded ‘yes’, a follow up question was asked: “Who treated you unfairly or discriminated against you because of your condition/s?” A list of multiple responses was provided, that consisted of employer, work colleagues, family or friends, teacher or lecturer, health staff (e.g., GP, nurse, hospital staff), bus drivers/rail staff/taxi drivers, restaraunt/hospitality staff, sales assistants, strangers in the street, or ‘others’.

Following these questions, respondents were asked “In the last 12 months have you avoided situations because of your condition(s)?” Again, those who responded ‘yes’ were asked the following question: “What situation(s) did you avoid because of your condition(s)?” A list of multiple responses was provided including work, visiting family or friends, school, university or educational facility, medical facilities (e.g., GP, dentist, hospital), shops, banks etc., restaurants, cafes or bars, public transport, public park or recreation venue, other social situations, other public places, other.

In addition to these new measures on discrimination, the SDAC instrument collected detailed measures of the recipient’s demographic characteristics and disabling conditions. Measured disabling conditions included sensory and speech, intellectual, physical, psychosocial, head injury and other. For the first time in 2015, the SDAC measured psychosocial disabilities. This concept is broader than a psychological disability and encompasses nervous or emotional conditions, memory problems or confusion, social or behavioral difficulties, a mental illness for which help or supervision is required and brain injuries including stroke, that lead to mental illness or other cognitive problems [14]. This concept was designed by the ABS to specifically measure conditions that “have an impact on a person’s ability to participate fully in daily living and opportunities like education, employment, and social and cultural activities” [14]. The ABS also included a measure of the number of long-term health conditions. Long-term health conditions defined by the ABS included diseases or disorders lasting or likely to last more than 6 months at the time of the survey.

### Statistical model

To model the association between demographic factors, health characteristics and the experiences of discrimination and avoidance, we fit logistic regression models. Using the raw logit coefficients, we calculate odds ratios (OR) which measure the change in the odds of experiencing discrimination or avoidance given a change in a covariate, once all other factors in the model are controlled.

Variable selection for all models was informed by the range of literature on discrimination outlined above. Model fit between alternative specifications was assessed using the Bayesian Information Criteria following Raftery’s procedure [16]. With all models specified, we checked the conditioning of the matrix of independent variables to investigate any collinearity influence [17]. The condition numbers and variance inflation factors (Mean VIF < 2) were very small providing support for the model specification. Final goodness-of-fit for the discrimination and avoidance models was confirmed using the Hosmer and Lemeshow test [18].

Due to the complex survey design, additional adjustments were necessary to generate correct variance estimates. Unfortunately, the ABS does not provide information on the primary selection unit due to privacy concerns. However, the ABS provides 60 replicate weights, in addition to a person weight, to adjust for sample design and non-response. Utilising an algorithm developed by Winter (2008), we employed the delete-one jackknife method to provide correct standard errors for the estimated logit coefficients [19, 20]. This provided an alternative to the standard Taylor series linearization methods when only replicate weights were available.

## Results

**Fig. 1:**
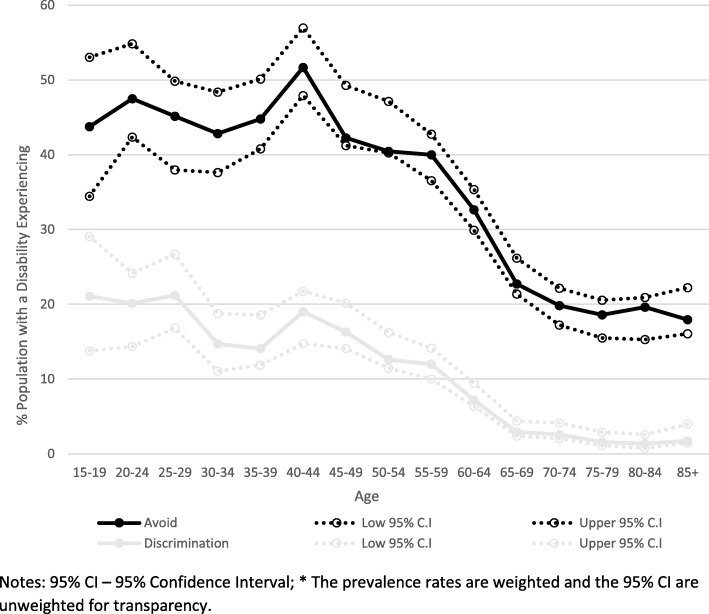
Prevalence of Discrimination (grey line) and Avoidance (black line) by Age, 2015*. Notes: 95% CI – 95% Confidence Interval; * The prevalence rates are weighted and the 95% CI are unweighted for transparency

In 2015, approximately 8.6% (95% CI = 8.1, 9.2) of people with a disability reported experiencing discrimination in the previous year. A considerably higher proportion, 31% (95% CI = 30.9, 32.9) reported an instance of avoidance due to an underlying disability. Viewing these prevalence rates across the life course, there was a clear age pattern in both perceived discrimination and avoidance. Prevalence rates were high between ages 15 to 44 years, declining from 45 to 65 years, and low and stable from 65 years onwards (Fig. 1). Indeed, from 65 years onwards, less than 2.5% of the population cited an experience of discrimination, compared with around 15% of those aged 15 to 29 years. Although cited discrimination was lower in later life, about 1 in 5 people aged 65 years and over actively avoided situations due to their disability.

However, age was only one characteristic by which discrimination and avoidance behaviours differed (Table 1). Table 1 displays the percentage of respondents reporting experiencing discrimination or avoidance behaviours, disaggregated by demographic and health factors. Across both discrimination and avoidance behaviours, prevalence appeared higher for those born in Australia (versus not), for those not married (versus married, with the exception of widowhood), for the unemployed (versus employed) and for those in the lowest 20% of the income distribution. Interestingly, there were no gender differences in the experiences of discrimination; however, females were more likely to cite avoidance behaviours than males. Similarly, those with higher levels of education were more likely to cite avoidance behaviours, but little differences were observed by discrimination. Region of residence appeared to have little bearing on the prevalence of either discrimination or avoidance.

**Table 1:** Prevalence of Discrimination and Avoidance by Demographic and Health Characteristics, 2015*

	Discrimination (%)		Avoidance (%)	
Demographic Factors				
Gender				
Male	8.3	–	28.2	–
Female	8.9		35.1	***
Country of Birth				
Australia	9.7	–	34.0	–
MESB	6.6	***	24.9	***
Other	5.2	***	27.3	***
Social Marital Status				
Married	5.4	–	26.9	–
Separated	16.9	***	37.0	***
Divorced	11.0	***	39.7	***
Widowed	2.9	***	21.9	***
Never Married	16.8	***	44.1	***
Region of Residence				
Major City	8.2	–	32.4	–
Inner Regional	9.4		31.6	
Other	9.4		29.9	*
Labour Force Status				
Employed	9.2	–	31.1	–
Unemployed	28.6	***	50.4	***
NILF	7.4	*	31.4	
Income Distribution				
0–20%	11.8	–	36.1	–
20–40%	8.9	***	33.2	*
40–60%	8.9	***	32.2	**
60–80%	7.5	***	28.5	**
80–100%	7.2	***	27.3	***
Not stated	6.8	***	30.0	***
Education				
Degree or above	9.0	–	35.1	–
Certificate	9.7		35.0	
School Only	7.7		29.0	***
Not Determined	10.1		29.2	***
Health Factors				
Number of Long Term Conditions				
1	7.0		24.9	
2	6.8		26.0	
3	8.7		30.4	
4	7.1		30.3	
5	9.8		35.1	
6	8.2		40.0	
7	10.6		43.0	
8	13.0		48.6	
9+	22.3		53.7	
	122.4	***	237.8	***
Disability Types				
No -Sensory/Speech	9.4	–	34.9	–
Yes -Sensory/Speech	6.9	***	25.3	***
No - Intellectual	7.6	–	30.5	–
Yes - Intellectual	21.0	***	49.1	***
No - Physical	7.2	–	24.5	–
Yes - Physical	9.2	***	35.1	***
No - Psychosocial	5.8	–	24.5	–
Yes - Psychosocial	21.6	***	65.5	***
No - Head Injury	8.2	–	30.9	–
Yes - Head Injury	15.5	***	47.0	***
No - Other	5.6	–	21.8	–
Yes - Other	12.4	***	44.8	***
Disability Severity				
Profound Limitation	12.5	–	52.4	–
Severe Limitation	13.0		51.9	
Moderate Limitation	10.3		39.1	***
Mild Limitation	6.5	***	21.9	***
Education Restrict	13.2		41.1	***
None	3.1	***	13.7	***

Notes: - comparison category for Wald tests of proportions; ***p < 0.001 **p < 0.01 *p < 0.05; MESB Main English Speaking Background countries

Of the health factors, a general pattern emerged whereby with an increasing number of long-term health conditions, the probability of experiencing discrimination or engaging in avoidance behaviours increased. For example, 22% of individuals with 9 or more long-term conditions reported experiencing discrimination, compared with 7% of people with just 1 long-term condition. Similarly, over half of all people with 9 or more multiple conditions reported avoidance behaviours compared with about one quarter of those with 1 long-term condition. Types of conditions also appeared to be associated with discrimination and avoidance. Those with an intellectual, physical, psychosocial or other disability were significantly more likely to report avoidance. When focusing on the severity of the disability, the results were more mixed. This may be associated with the age prevalence rates of differing disabling conditions.

Although these differences in the prevalence of discrimination and avoidance behaviours are informative, the associations may be confounded by unobserved factors. For example, does the different age profile of those born overseas account for a lower probability of discrimination or avoidance? Are more highly educated individuals more likely to cite an experience of discrimination because of age cohort differences in educational attainment? Results in Table 2 show odds ratios, measuring the odds of experiencing discrimination or avoidance behaviours for a range of demographic groups with different health characteristics, once all other factors in the model were controlled. In assessing model fit, Bayesian Information Criterion tests confirmed a model of disability types over disability severity, and a model including labour force status rather than income.

**Table 2:** Logistic Regression Models of Disability Discrimination and Avoidance, 2015

	Discrimination OR		Avoidance OR	
Age				
15–29	–		–	
30–44	0.63	**	0.85	
45–59	0.46	***	0.62	***
60–74	0.14	***	0.31	***
75–84	0.05	***	0.23	***
85+	0.05	***	0.19	***
Gender				
Male	–		–	
Female	0.99		1.32	***
Country of Birth				
Australia	–		–	
MESB	0.99		0.78	**
Other	0.67	*	0.78	*
Social Marital Status				
Married	–		–	
Separated	1.88	**	0.91	
Divorced	1.42	*	1.25	**
Widowed	1.22		1.04	
Never Married	1.29		1.08	
Region of Residence				
Major City	–		–	
Inner Regional	1.15		0.94	
Other	1.20		0.91	
Labour Force Status				
Employed	–		–	
Unemployed	2.43	***	1.46	*
NILF	1.06		1.19	*
Education				
Degree or above	–		–	
Certificate	0.82		0.84	
School only	0.74	*	0.63	***
Undetermined	1.03		0.65	*
Number of LTC	1.16	***	1.12	***
Disability Type				
Sensory & Speech	1.33	**	1.10	
Intellectual	1.35	**	1.09	
Physical	1.37	**	1.64	***
Psychosocial	1.99	***	3.74	***
Head Injury	0.78		0.72	*
Other	1.81	***	2.21	***

Notes: - comparison category; ***p < 0.001 **p < 0.05 *p < 0.1; MESB Main English Speaking Background Countries; NILF Not in the Labour Force

For both discrimination and avoidance models respectively, risk was higher for people of younger ages (85+ OR = 0.05 *p* < 0.01, 85+ OR = 0.19, *p* < 0.01), for divorced individuals (OR = 1.42 *p* < 0.05, OR = 1.25 *p* < 0.01) and unemployed individuals (OR = 2.43 *p* < 0.001, OR = 1.46 *p* < 0.05.) Interestingly, lower educated people were less likely to cite discrimination (OR = 0.74 *p* < 0.05) or avoidance (OR = 0.63 *p* < 0.05). Consistent with the descriptive results, females were more likely than males to cite avoidance (OR = 1.32 *p* < 0.001), but no more likely to report experiencing discrimination because of their disability when compared to males (OR = 0.99 *p* > 0.1).

Controlling for these important demographic characteristics, measures of health and disability remained strongly associated with discrimination and avoidance. People with a psychosocial (OR = 1.99 *p* < 0.001, OR = 3.74 *p* < 0.001), physical disability (OR = 1.37 *p* < 0.001, OR = 1.64 *p* < 0.001) or other disability (OR = 1.81 *p* < 0.001; OR = 2.21 *p* < 0.001) were considerably more likely to cite instances of discrimination or avoidance, relative to those without these disability types. Independent of specific disability types, for each increase in the number of long-term conditions a person has, the incidence of discrimination and avoidance increased by 1.16 (OR = 1.16, *p* < 0.001) and 1.12 (OR = 1.12, *p* < 0.001) respectively. Although not statistically significant in explaining avoidance, those with a sensory and speech or intellectual disability were more likely to experience discrimination when compared to those without these disabilities.

In addition to highlighting groups at a higher risk of avoidance and discrimination, the SDAC data enabled an examination of the context of these experiences due to disability. Results in Table 3 show the types of situations avoided and sources of discrimination for those who reported either experience (Table 3). Among the sources of discrimination, employer (24%), family or friends (23%), strangers in the street (21%) and other (26%) were the most heavily cited. Importantly, about 18% of those who experienced disability discrimination cited the source of discrimination as a health worker. As expected, there was significant variation in the sources of discrimination by age. Younger and working age Australians were more likely to cite employer or work colleagues and were more likely to cite a higher number of discrimination sources than older people.

**Table 3:** Context of Disability Discrimination and Avoidance, 2015

	15–29		30–44		45–59		60–74		75–84		85+		Total
Source of Discrimination (%)													
Employer	30.0	–	39.2		21.0	*	6.2	***	0.0	***	0.0	***	24.0
Work colleagues	23.2	–	16.2		16.0		7.6	***	0.0	***	0.0	***	15.4
Family or friends	29.5	–	26.0		21.8		15.6	**	31.0		8.6		23.3
Teacher or lecturer	17.7	–	3.4	***	3.3	***	0.0	***	0.0	***	0.0	***	5.2
Health staff (GP, nurse, hospital staff)	17.3	–	16.6		17.7		20.1		18.8		12.9		17.7
Bus drivers/ rail staff/ taxi drivers	3.4	–	3.0		3.1		7.2		4.4		4.7		3.9
Restaurant/ hospitality staff	5.1	–	7.7		3.2		5.7		4.4		9.4		5.2
Sales assistants	11.9	–	8.7		10.9		12.7		12.9		0.0	***	10.7
Strangers in the street	21.9	–	21.7		18.7		20.4		37.2		23.7		20.9
Other	20.7	–	22.0		27.4		33.5	*	17.7		45.3		25.8
Number of Discrimination Sources	1.8	–	1.6		1.4	***	1.3	***	1.3	*	1.1	**	1.5
Situations Avoided (%)													
Work	33.2	–	41.4		30.9		10.4	***	2.1	***	1.3	***	24.1
Visiting family or friends	42.0	–	48.6		43.8		33.4	*	25.3	***	27.0	***	39.5
School, University or educational facility	22.5	–	11.4	***	8.8	***	3.5	***	2.1	***	0.0	***	8.5
Medical facilities (GP, dentist, hospital)	17.9	–	16.0		10.9	**	6.8	***	4.7	***	5.5	***	10.8
Shops, banks etc.	34.1	–	37.1		36.2		26.8		29.6		33.2		33.0
Restaurants, cafes or bars	31.9	–	33.0		32.2		26.0		29.8		34.6		30.6
Public transport	22.6	–	23.5		24.8		24.2		25.7		37.1	*	24.7
Public park or recreation venue	16.5	–	22.5	*	19.0		17.4		20.4		19.0		19.1
Other social situations	57.7	–	56.8		50.3	*	42.3	***	41.5	***	41.7	**	49.2
Other public places	25.5	–	28.2		27.7		27.6		24.5		25.3		27.2
Other	12.1	–	9.9		9.9		15.8	*	12.6		13.1		12.1
Number of Situations Avoided	3.2	–	3.3		2.9	*	2.3	***	2.2	***	2.4	***	2.8

Notes: - comparison category for Wald tests of proportions; ***p < 0.001 **p < 0.01 *p < 0.05. % weighted percentages

Of the 31% of individuals with a disability who reported avoidance, about half avoided ‘other social situations’ (49%). Over 30% avoided visiting family or friends (39.5%), shops and banks (33%) and restaurants, cafes and bars (30.6%). Importantly, about 11% of respondents with a disability actively avoided medical facilities, and the prevalence of this form of avoidance was much higher among younger and middle-aged Australians (16–18% for 30–44 year olds and 15–29 year olds respectively). From a human capital perspective, it is also important to note the high prevalence of avoidance of education facilities (22.5 and 11.4%) and work (33.2 and 41.4%) by these same age groups. Older Australians tended to cite a lower number of avoidance situations relative to younger Australians. However, there appeared to be no significant age-related pattern of avoidance of shops and banks, restaurants, cafes and bars, public park or recreation venues or other public places. Those in the oldest age group (85+ years), cited particularly high levels of avoidance of public transport (37%).

## Discussion

Despite protections for people with a disability against exclusion enshrined in legislation, results from this study show that over a 12-month period, a significant proportion of respondents reported experiencing an instance of discrimination (8.6%) or avoidance (31.0%). These results confirm those previously described by the Australian Bureau of Statistics, but are slightly lower than those reported in an analysis of disability discrimination limited to the working age population (persons aged 15–64 years) [12]. The slightly lower prevalence that we observe, consistent with the ABS, is due to the significantly lower prevalence of discrimination in later life (after age 65 years) which we address further in this paper.

### Avoidance and discrimination

Before turning to the key research questions of this study, it is useful to consider potential exlpanations for the higher prevalence of avoidance relative to discrimination among Australians living with a disability. Conceptually, avoidance is a broader concept than discrimination as it may capture functional limitations in engaging in certain contexts or situations. For example, facing access difficulties to public places due to mobility issues. However, the Australian Government through the Australian Human Rights Commission (AHRC) considers such avoidance behaviours to be a measure of “indirect discrimination”. Specifically, they argue:

“Discrimination can be direct, meaning a person with a disability is treated less favourable than a person without that disability in the same or similar circumstances… Indirect discrimination can happen when conditions or requirements are put in place that appear to treat everyone the same, but actually disadvantage some people because of their disability. For example, it may be indirect discrimination if the only way to enter a shop is by a set of stairs, because people with disability who use wheelchairs would be unable to enter the building.” [21].

This form of indirect disability discrimination through avoidance behaviours has rarely been examined using nationally representative data. It highlights an important measurement issue in that measuring disability discrimination alone without measures of avoidance may provide considerably lower point estimates of exclusion more generally. Indeed, the extant literature provide important evidence of the link between experiences of discrimination and avoidance behaviours. For example, empirical studies show people who experience disability discrimination are less likely to utilise health services [22–24], less likely to access preventative health services, delay or fail to fill prescriptions and delay or avoid treatment [24, 25]. This avoidance behaviour can contribute to the development of undiagnosed comorbidities, further increasing the burden of poor physical and mental health upon the individual, community and economy [24].

### Variations in discrimination and avoidance by demographic characteristics

Given the relatively high levels of avoidance and sizeable minority of people with a disability reporting discrimination, this paper sought to firstly examine whether particular demographic groups were at a heightened or reduced risk of exposure, once controls for health factors were included. Although descriptive statistics have been released elsewhere, this study examined the unique contribution of a number of demographic variables to discrimination, once all factors included in the multivariate analyses were controlled for. With extensive controls for disability and demographic factors and adjusting for complex survey design, we confirm that disability discrimination and avoidance is spread unevenly throughout the population. Notably, we find that disability discrimination and avoidance decreases with age. A speculative explanation is that as the prevalence of disability increases with age, it becomes more normative and therefore socially acceptable. In contrast, disabilities are less common amongst younger people and therefore more likely to be a point of differentiation and discrimination. This hypothesis of discriminatory attitudes stems from the social psychology Social Identity Theory of the ‘in’ versus ‘out’ group mentality, developed by Tajfel and Turner in 1979 [26]. Social Identity Theory leads to intergroup social comparison where individuals in one group compare themselves against their out-group [27]. In-group versus out-group comparison frequently creates tension, conflict or discrimination. Based on an individual’s perceived commonality with other group members, one might have several group-memberships [28]. As the likelihood of gaining one or more disabilities increases as people age, more older people will self-identify within this group, thus mitigating any prejudice or discrimination.

Notably, avoidance issues were also found to decline with increasing age, reflecting several possible reasons. Firstly, Australians who are 65 years and older or 50 years and older for Aboriginal and Torres Strait Islander people, have access to the Home and Community Care program (HACC). Within this program individuals are able to access services such as nursing and allied health services, household domestic assistance and personal care thereby offsetting some of the issues that may make access to healthcare difficult and costly for younger cohorts. In addition, lower levels of perceived discrimination are also likely to be a driver of lower levels of avoidance.

There are also methodological issues which may effect the age grading of discrimination and avoidance that we observe here. Specifically, the cross-sectional design of SDAC enforces an important limitation on correctly identifying age differences in the reporting of discrimination and avoidance, particularly in the later lifecourse. As is well established in the economics of ageing literature, selective mortality removes a disproportionate number of older people with low levels of resources and poorer health from the population [29, 30]. Moreover, this selectivity effect may also operate through migrating out of the survey population through entry to aged care [29], a point that is particularly pertinent given that the SDAC module on discrimination did not include people in non-private dwellings. Longitudinal data is required to measure complex movements out of private households and further data collections would be necessary to examine the generalisability of these findings presented herein to individuals living in care accommodation and other institutions (non-private dwellings).

Once controls for age are included in the multivariable regression analyses, the likelihood of exposure to avoidance and discrimination was higher for divorced people (versus married), the unemployed (versus employed) and was lower for people with lower levels of education (versus a degree) and those born overseas. Interestingly, the broader literature on discrimination shows that demographic characteristics are associated with exposure to discrimination. For example, findings of fewer reports of discrimination among lower educated people has been cited in racial discrimination research. Cunningham and Paradies (2013) provide several reasons, specifically for education: “more educated Indigenous people (1) may have higher expectations about how others should treat them, a difference in interpretation rather than exposure; (2) are more likely to work and socialise with non-Indigenous people and hence be exposed to inter-racial discrimination … and (3) are more likely to be the targets of discrimination because they defy stereotypes” [31]. These explanations are likely to be relevant to an association between education and disability discrimination that we observe herein. Moreover, there is a considerable body of literature on the role of disability based discrimination and employment, underscoring the higher prevalence of discrimination among the unemployed. Levels of discrimination cited by the unemployed are likely partially reflective of discriminatory practices in recruitment processes that are oftentimes faced by people with disabilities [32].

### Variations in discrimination and avoidance by disability characteristics

With controls for demographic characteristics included, results from the multivariate regression analyses showed having a psychosocial or physical disability significantly increased the odds of experiencing discrimination or avoidance, as did an increasing number of long-term health conditions.

The findings that people with a psychosocial disability are more likely to report discrimination or avoidance is consistent with a large body of literature on mental health stigma more generally. For example, using Danish data, Dammeyer and Chapman (2018) find that those with a mental health disability were more likely to report both discrimination and violence [33]. More broadly, a recent systematic review of 144 studies has found that mental health related stigma is associated with avoidance of health seeking [34]. Indeed, it may be that individuals with psychosocial disabilities in particular, may be at greater risk of discrimination and avoidance due to mental health stigma. Stigma is based on negative attitudes and misunderstanding that leads to prejudice and discrimination. Stigma can result in a lack of support and empathy for individuals living with a mental illness, which can leave sufferers feeling embarrassed, misunderstood, and marginalised. Stigma may affect self-esteem, leading to avoiding seeking treatment, and the social withdrawal from the rest of the community through avoidance [35].

Our study further shows that respondents with a physical disability were more likely to cite both discrimination and avoidance, relative to those without this disability. We speculate that physical disability is more ‘visible’ relative to other health conditions, creating a point of difference to the norm and consequently a visible target for discrimination. Several studies have shown that individuals with high visible disabilities are treated less favourably than people with less visible or invisible disabilities. For example, Gouvier et al. 1991 showed that job applicants with visible disabilities received the most unfavourable ratings by potential employers. This was particularly the case in recruiting for jobs requiring high levels of public contact [36]. Similarly, Crocker and Major, 1994 noted individuals with invisible disabilities had less problematic or anxiety-provoking social interactions than people with more visible disabilities [37].

More generally, people with physical disabilities may avoid social, familial and economic situations due to physical and organisational obstacles such as inadequate access to transportation and a lack of communication assistance as well as discriminatory attitudes [38]. A recent international study shows that the association of discrimination with healthcare avoidance was particularly pronounced amongst people with physical disabilities, relative to those with other disability types [24]. This is important as studies show that avoidance behaviours, particularly when occurring in contexts important to human capital such as healthcare, is strongly associated with experiencing psychological distress [11]. Moreover, there is new evidence that the ‘effect’ of disability discrimination and avoidance on poor mental health outcomes is exaserbated for respondents living with physical disabilities including incomplete use of arms, fingers or legs and feet or those with experiences of blackouts/seizures [39].

### Context of Disabilitiy discrimination and avoidance

Our final research objective was to understand the experience of discrimination and avoidance in different settings and contexts across an individual’s lifecycle. Importantly, we demonstrated that disability discrimination and avoidance occurs in contexts that are vital to human capital investments. For example, in healthcare settings, education settings and within the labour force. Consistent with a lifecycle approach, discrimination and avoidance in the workplace and education settings were prominent among younger and middle age Australians with a disability.

Indeed, both avoidance and discrimination in the workplace specifically due to physical barriers has been cited by the Australian Human Rights Commission, who note mobility and access issues combined with an unwillingness of employers to implement reasonable adjustments to the physical environment [21]. Labour force participation of people with a disability is 54% compared with 82% for people without a disability and this ratio has been stable over the past decade [40]. The Australian Human Rights Commission’s 2016 National Inquiry report cited several brief case studies indicating specific failings of reasonable workplace adjustments and the barriers that such failure makes to on-going employment for people with disabilities [32], pp. 187–191]. This is despite the fact that many benefits of employing people with disability were identified in an earlier report [41].

Further work by Darcy, Taylor and Green (2016) using natural data from the Australian Human Rights Commission determined that disability discrimination complaints comprised the largest proportion of cases compared with all other discrimination types in Australia [42]. Within the disability discrimination complaints data, employment discrimination made up the greatest proportion in these cases. They found that people of employment age with physical mobility disability reported higher rates of discrimination, particularly in relation to access barriers in the workplace. They speculate that employers incorrectly assume expensive hiring costs associated with potential employees who have a disability and may also be unaware of government subsidy programs to assist in making workplace adjustments.

We further identified experiences of discrimination and avoidance due to disability in education settings, particularly among younger Australians. The existing literature notes that students living with disability are often excluded and/or teachers refuse to or are reluctant to make reasonable adjustments [43]; amounting to institutional discrimination [44]. A Senate Inquiry in 2016 received evidence of systemic barriers experienced by students with disability and their families including “difficulties enrolling, failure of schools to provide the reasonable adjustments required by students, exclusion from school activities, a shortage of services in rural and remote areas of Australia and low expectations of students with disability from school staff and others, leading to a failure to take seriously the educational needs of students” [45]. This was compounded by the family’s financial means, geographical location and indigenous status.

Interestingly, whereas education and workplace discrimination and avoidance was clustered among younger and middle aged Australians, the same was not the case for discrimination in healthcare settings. The likelihood of discrimination in healthcare was relatively flat, or invariant, by age. Discrimination in healthcare settings is particulary important as recent research shows disability discrimination specifically, may be associated with deteriorating health as well as avoidance of healthcare services [24, 46]. Experiencing access barriers to healthcare, such as discrimination, can demotivate people to attend to current illnesses posing further issues for individuals’ re-engagement into effective healthcare treatment. Delaying treatment for deleterious health conditions may lead to an escalation in illness severity, contributing to a greater health burden [47, 48]. This may contribute to poorer quality of life, unnecessary hospitalisations and eventually placing greater strain on acute care systems [49–51].

### Limitations

In interpreting the results from this study, it is important to note the limitations. Firstly, the data are cross-sectional and as noted earlier, there is a possibility of a selectivity effect in survival. As individuals with higher economic and social resources are more likely to exhibit higher survival prospects relative to their financially disadvantaged peers; in cross-sectional data we may be observing these individuals [52]. Moreover, the SDAC module on discrimination was only completed by repondents living in private households in the community. Further data collections would be necessary to generalise these findings to persons with a disability living in non-private dwellings such as those in long-term care residences. Longitudinal data would be required to disentangle these effects, unfortunately there is a dearth of information on the experiences of disability discrimination in Australia. In addition, the measures utilised by the ABS rely upon recall over a 12-month period, and thus may be subject to recall bias. The measures on discrimination are also self-reported and may be subject to further bias. For example, some individuals may feel uncomfortable disclosing or discussing such experiences, biasing the prevalence downwards.

### Recommendations for further research

Noting these limitations, the levels of disability discrimination and avoidance that we observe pose questions about the implications for the individual. In the broader field of research in social inequalities in health, pathways between exposure to discrimination and poor health outcomes (both physical and mental), have been extensively investigated specifically in the field of racism research [53]. Racism has been shown to be associated with negative health impacts through several key pathways including by: (1) increasing stress; (2) decreasing health promoting behaviours (e.g. physical activity); (3) increasing health-damaging behaviours (e.g. alcohol and drug use); (4) reducing access to key health-promoting resources (e.g. employment, education, health and aged care services etc.); (5) increasing dysregulation (e.g. sleep disruption); and (6) increased health-damaging exposures (e.g. toxic substances) [53]. The results may also potentially relate to the increased levels of exposure to other types of discrimination, including for people with disabilities and the variability of these exposures over the life span and with demographic characteristics [54, 55]. Recent Australian studies have provided some evidence that experiencing discrimination is associated with poor mental health outcomes and lower levels of self-rated health [11, 12, 39]. However, an important area of future research is to validate the pathways between exposure to disability discrimination and deleterious health outcomes, as has occurred in racism research.

Related to that above, the second key research priority is the use of longitudinal data with validated measures of disability discrimination and avoidance. The cross-sectional results from SDAC, although informative, are limited to measuring a statistical association, rather than enabling a determination of more complex causal pathways between disability exclusion and health outcomes. In the Australian case, further data collections are necessary.

Although not the focus of this paper, the high prevalence of discrimination and avoidance reported by some groups of people living with disabilities and in specific contexts, suggests the time is ripe for policy overhaul and immediate attention. In particular, through the continued roll out of the National Disability Insurance Scheme (NDIS), it is important to consider the role of disability exclusion in acting as a barrier to accessing mainstream services for people living with a disability.

Many policy solutions have been offered in the literature. For example, positive media campaigns and general education by way of interventions are an obvious way to address physical and psychosocial disability stigma and discrimination. Beyondblue, an Australian NGO, suggests two effective approaches to reduce stigma: (i) educational approaches which involves the dissemination of factual information through media, social media, books, flyers, movies, websites etc. and (ii) contact approaches which involves direct interpersonal contact with people with a psychosocial disability [35]. However, consideration must be given to strategies that are delivered in a collaborative, sustainable and multi-sectoral way and be supported by reform and policies that influence national attitudes and behaviours [35].

Apart from these general recommendations, the heterogeneity in discrimination and avoidance that we observe in contexts critical to human capital (eg., healthcare, education, workplace) strongly underscores the need for sectoral solutions. For example, the 2016 Senate Inquiry received evidence of systemic barriers experienced by students with disability and their families [45]. They concluded with ten comprehensive recommendations which included the establishment of a national strategy around driving a cultural change, particularly at leadership level, to recognise all students with disability as learners; increase school participation and access rates for students with disability; ensure students with disability can access adjustments and interdisciplinary support to maximise learning; improve accountability; and establish an independent review and complaints mechanisms so all stakeholders can have full confidence in the system.

Strong support for addressing discrimination in employment have also become a national priority as a results of the Australian Human Rights Commission’s *Willing to Work* Inquiry [32]. Consequently, recommendations from this Inquiry included supporting increasing the workforce participation of people with disabilities by (i) forming a national priority around the issue, (ii) which is closely linked with policy planning for an ageing workforce and (iii) in conjunction with goals to reduce disability stigma and avoidance.

Although sectoral responses to disability discrimination and avoidance are now being considered by governments as indicated above, there is considerable opportunity for research to help direct implementation and evaluate proposed strategies.

## Conclusion

Noting these limitations and extensions, results from this study show that discrimination (8.6%) and avoidance (31%) due to disability in Australia is prevalent, is higher in certain demographic groups within the population (such as younger people and the unemployed), and is more prevalent among individuals with specific disability types (physical and psychosocial). We further show that experiences of disability discrimination and avoidance does occur in contexts crucial to human capital investments, following the human lifecycle through education, labour market and healthcare contexts. With reforms under way through the NDIS and prioritisation of the employment of people with a disability by the Australian government, addressing discriminatory behavior and practices at a sectoral level is critical in realising the goals of this reform agenda. Irrespective of its consequences, disability discrimination is a violation of human rights and should be prevented in any reasonable society. With inexorable population ageing increasing the numbers of people living with a disability, the urgency to address disability discrimination is considerable.

## Abbreviations

ABS: Australian Bureau of Statistics
AHRC: Australian Human Rights Commission
CRPD: Convention on the Rights of Persons with Disabilities (CRPD)
GP: General Practitioner
HAAC: Home and Community Care program
NDIS: National Disability Insurance Scheme
NGO: Non-Government Organisation
OR: Odds Ratios
SDAC: Survey of Disability, Ageing and Carers
UK: United Kingdom
UN: United Nations
VIF: Variance Inflation Factors
